# Vitamin B12 Deficiency Associated with Metformin and Proton Pump Inhibitors and Their Combinations: Results from a Disproportionality and Interaction Analysis

**DOI:** 10.3390/diseases13100334

**Published:** 2025-10-10

**Authors:** Kannan Sridharan

**Affiliations:** Department of Pharmacology & Therapeutics, College of Medicine & Health Sciences, Arabian Gulf University, Manama P.O. Box 26671, Bahrain; kannans@agu.edu.bh

**Keywords:** metformin, pantoprazole, rabeprazole, esomeprazole, vitamin B12 deficiency

## Abstract

Background: Metformin and proton pump inhibitors (PPIs) are independently associated with vitamin B12 deficiency. Despite frequent co-prescription, particularly in diabetics with gastroesophageal disorders, evidence regarding the combined risk of these medications on vitamin B12 deficiency remains limited. This study aimed to evaluate the real-world risk of vitamin B12 deficiency associated with metformin, PPIs, and their combinations using the United States Food and Drug Administration Adverse Event Reporting System (USFDA AERS) database. Methods: We conducted a disproportionality analysis using USFDA AERS data from 2004 to 2024. We assessed whether metformin, PPIs, or their combinations were reported more often than expected with vitamin B12 deficiency and evaluated associated clinical outcomes, such as hospitalization and life-threatening events. Results: Among 29,661,136 reports, 552 met inclusion criteria, with metformin monotherapy accounting for 274 cases. Positive safety signals were detected for both metformin and all PPIs individually. While statistical interaction measures were not conclusive, patients on metformin–pantoprazole combination therapy experienced significantly higher rates of hospitalization and life-threatening events compared to those on pantoprazole alone. Conclusions: These findings suggest that patients receiving metformin and PPIs together, particularly the elderly, may face a higher risk of serious vitamin B12 deficiency-related complications. Clinicians should consider closer monitoring of vitamin B12 levels and supplementation when needed in patients on combination therapy.

## 1. Introduction

Metformin remains the cornerstone of oral antidiabetic therapy, prescribed to nearly 50% of diabetic patients worldwide, with an estimated 150 million users annually [1]. Utilization rates exceed 80% even in the presence of contraindications, underscoring its widespread use [2]. Current treatment guidelines consistently recommend metformin as the first-line therapy for type 2 diabetes, with exceptions mainly for patients with chronic kidney disease and heart failure [3]. Among its adverse effects, vitamin B12 deficiency is of particular concern, especially with long-term use, where prevalence may reach up to 50% [4]. The proposed mechanisms include altered intestinal motility with bacterial overgrowth, impaired intrinsic factor-mediated absorption, and calcium-dependent interference with uptake [5]. Clinically, deficiency manifests as megaloblastic anemia and peripheral neuropathy, both of which can substantially impair quality of life [6]. Vitamin B12 deficiency can lead to a spectrum of clinical manifestations, ranging from fatigue, lethargy, and pallor to neurological symptoms such as numbness, tingling, gait disturbances, and cognitive impairment. In severe cases, it can result in megaloblastic anemia, peripheral neuropathy, and irreversible neurological damage. These symptoms significantly impact patients’ quality of life and may be easily overlooked, particularly in elderly populations where they can be mistaken for normal aging or comorbid conditions.

Proton pump inhibitors (PPIs), widely prescribed for gastroesophageal reflux disease and peptic ulcer, reduce gastric acid secretion through irreversible inhibition of proton pumps [7]. Diabetic patients are more likely to require PPIs, given their elevated risk of gastroesophageal disorders (OR: 1.6; 95% CI: 1.4, 1.9) [8]. Long-term PPI therapy has been linked to vitamin B12 deficiency, primarily via hypochlorhydria, with deficiency rates reported up to 54.12% of omeprazole users and 45.87% of pantoprazole users [9]. While some studies in older adults found no association [10], pooled analyses confirm a significant relationship (OR: 1.42; 95% CI: 1.16, 1.73) [11].

This issue carries notable clinical significance. Gastroesophageal reflux disease affects 40% of patients with type 2 diabetes, and 70% of these individuals receive oral antidiabetic therapy [12]. Consequently, metformin and PPIs are frequently co-prescribed, raising concern for additive or synergistic risk of vitamin B12 deficiency. Reported prevalence rates support this, with 21.91% in metformin monotherapy, 25.58% in PPI monotherapy, and 34.15% in combination therapy [13]. Yet, despite these figures, comparative safety data on combination therapy versus monotherapies remain limited.

The United States Food and Drug Administration Adverse Event Reporting System (USFDA AERS) provides an opportunity to explore this gap through pharmacovigilance signal detection. Disproportionality analysis of such real-world data enables the identification of rare adverse events, including vitamin B12 deficiency [14,15]. This study therefore aims to assess the association between metformin, PPIs, and their combination with vitamin B12 deficiency using the USFDA AERS, with particular emphasis on whether co-prescription amplifies risk beyond that observed with either drug alone.

## 2. Methods

### 2.1. Data Source

The primary data source for this investigation was the USFDA AERS database. We systematically extracted data using specific MedDRA (Medical Dictionary for Regulatory Activities) Preferred Terms (PTs) related to vitamin B12 deficiency, including:

- “Vitamin B12 deficiency” (MedDRA code: 10047609)
- “Vitamin B12 decreased” (MedDRA code: 10047608)
- “Anaemia vitamin B12 deficiency” (MedDRA code: 10002080)
- “Neuropathy vitamin B12 deficiency” (MedDRA code: 10079953) [16]

The temporal scope of data collection encompassed 83 quarterly reports, spanning from the first quarter of 2004 through the third quarter of 2024, ensuring a comprehensive longitudinal analysis of adverse event patterns.

### 2.2. Data Processing

The USFDA AERS was systematically searched for reports involving metformin and PPIs and the combinations of PPIs with metformin to ensure comprehensive retrieval of Individual Case Safety Reports (ICSRs). The following PPIs were included in this study: pantoprazole, omeprazole, esomeprazole, rabeprazole, lansoprazole and dexlansoprazole. The search strategy is outlined in Electronic Supplementary Table S1. We adhered to the USFDA’s deduplication guidelines, sorting reports in ascending order by Case_IDs and retaining only those with the latest FDA_DT or Individual Safety Report number, representing the most recent entry. Following this, a manual check was carried out and additional duplicate reports were identified based on the date of receiving the reports, reporting country, age, gender, indications and outcome. Our search was limited to generic names of metformin and the selected PPIs. From the deduplicated reports, we extracted key variables including patient age, sex, year of report, and the country of origin for cases involving metformin, PPIs, or their combination therapies.

### 2.3. Data Mining Algorithms

We applied a ‘case–non-case’ disproportionality analysis to assess the relationship between metformin (alone or in combination with PPIs) and the specified PTs by comparing the frequency of vitamin B12 deficiency reports associated with metformin. PPIs or their combinations with reports involving all other drugs [17]. Data retrieval and analysis were conducted using the OpenVigil 2.1 platform. We used two frequentist and two Bayesian data mining algorithms to detect potential safety signals with vitamin B12 deficiency.

In the frequentist approach, we calculated the Reporting Odds Ratio (ROR) and the Proportional Reporting Ratio (PRR). The ROR is the ratio of odds ratio of reporting vitamin B12 deficiency with the drug/s of interest upon the odds ratio of reporting the same adverse event with all other drugs. The PRR is the ratio of proportion of reporting vitamin B12 deficiency with the drug/s of interest upon the proportion of reporting the same adverse event with all other drugs. Signal detection followed the criteria established by Evans, which require at least three reports, a PRR greater than 2, and a chi-square (χ^2^) value exceeding 4 for each drug–event pair [18]. For both ROR and PRR, 95% confidence intervals (CIs) were calculated, with a signal considered present if the lower bound of the ROR CI was above 1. Additionally, the Relative Reporting Ratio (RRR) was computed by dividing the observed number of cases by the expected number, providing a comparative measure of vitamin B12 deficiency associated with metformin, PPIs, or their combinations relative to the entire database.

Bayesian analyses were performed using the Bayesian Confidence Propagation Neural Network (BCPNN) and the Multi-Item Gamma Poisson Shrinker (MGPS). For BCPNN, signals were identified based on the Information Component (IC), calculated as the logarithmic ratio of the observed co-occurrence of metformin, PPIs, or their combinations with vitamin B12 deficiency compared to the expected frequency in the database. An IC-based signal was detected if the lower bound of the 95% CI (IC025) exceeded zero. MGPS used the Empirical Bayes Geometric Mean (EBGM), with a signal detected if the lower bound of the EBGM’s 95% CI (EBGM05) exceeded 2 [19].

It is important to note that analyses based on the FAERS database are inherently limited by well-recognized biases, including under-reporting, selective reporting peaks following safety alerts, absence of true denominators for calculating incidence, and lack of detailed clinical information such as dose, duration of therapy, comorbidities, and laboratory confirmation. These constraints mean that disproportionality signals reflect reporting patterns rather than definitive causal associations. Therefore, our findings should be interpreted as hypothesis-generating, requiring confirmation in well-designed observational or prospective studies.

### 2.4. Calculation of Interaction Signal Scores

The potential interaction between metformin and PPIs was assessed using Interaction Signal Scores (INTSS). These scores were derived from the EBGM values observed versus expected reports of vitamin B12 deficiency and calculated using the 90% confidence intervals (EB05 and EB95). The EB05 and EB95 values were determined separately for metformin and PPIs individually, as well as for their combined use [20]. INTSS was calculated as follows:

$$\frac{EB05\;of\;metformin\;with\;PPIs\;with\;vitamin\;B12\;deficiency}{Highest\;EB95\;of\;metformin\;or\;PPIs\;concerned\;for\;vitamin\;B12\;deficiency}$$

An INTSS >1 indicated a statistically significant interaction between metformin and the co-administered PPI regarding the risk of vitamin B12 deficiency [20].

### 2.5. Outcomes Assessed

The main outcomes assessed for metformin, PPIs, and their combination in relation to vitamin B12 deficiency included life-threatening events, disability, and both initial and prolonged hospitalizations.

### 2.6. Compliance with Reporting Standards

This study was conducted in accordance with the Reporting of a Disproportionality Analysis for Drug Safety (READUS-PV) guidelines for pharmacovigilance studies utilizing spontaneously reported adverse events [21].

## 3. Results

### 3.1. Search Results

**Figure 1:**
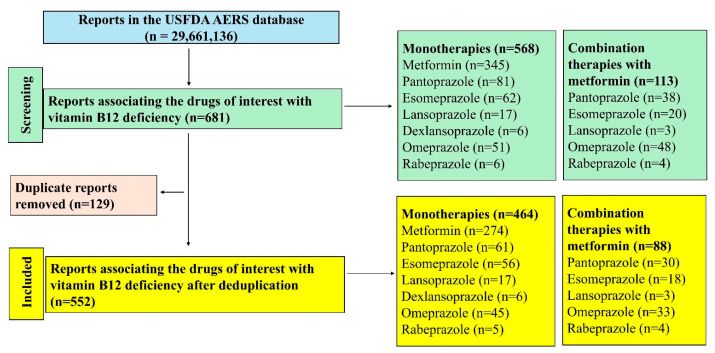
Study flow diagram. A total of 552 reports were included in this study.

A total of 29,661,136 reports existed in the database during the study period of which 552 were included in the final analysis (Figure 1). Metformin had the maximum number of reports (*n* = 274) followed by pantoprazole (*n* = 61) amongst the monotherapies and amongst the combinations, metformin with omeprazole (*n* = 33) had the most reports associated with vitamin B12 deficiency.

The demographic characteristics of the patients included in the reports are summarized in Table 1. Most patients were in the elderly age group (≥65 years) with no clear sex predilection observed.

### 3.2. Signal Detection Measures

**Figure 2:**
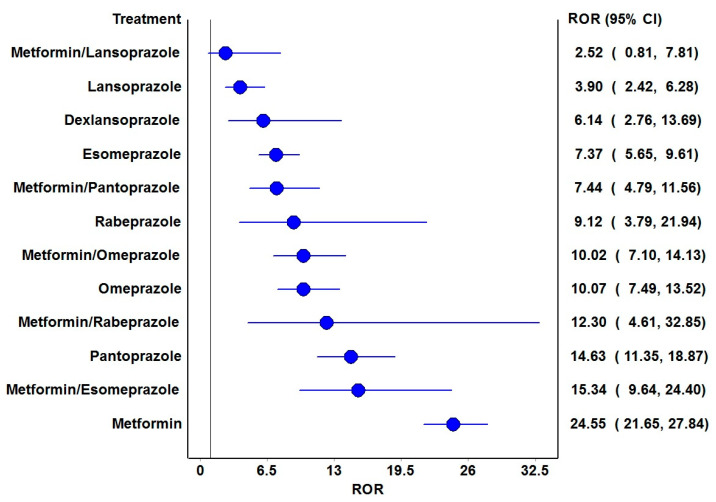
Reporting odds ratios of the drugs and their combinations. ROR: Reporting odds ratio. The blue circles represent the ROR, and the blue horizontal lines represent 95% CI of RORs. The vertical black line represents the line of no difference in the risk of vitamin B12 deficiency.

The frequentist and Bayesian signal detection measures are summarized in Table 2. Metformin and all PPIs have been observed with positive signals with the risk of vitamin B12 deficiency. Amongst the PPI combinations with metformin, pantoprazole, omeprazole, esomeprazole and rabeprazole generated positive signals by both approaches. The RORs of various drugs and their combinations are depicted in Figure 2 and all except metformin with lansoprazole were associated with significantly higher reporting odds.

### 3.3. Interaction Signal Scores

The INTSS for the combinations of metformin with PPIs is shown in Table 3. None of the evaluated combinations of metformin and PPIs were observed with significant interaction scores. However, with metformin combinations, esomeprazole and rabeprazole showed higher EBGM values compared to their monotherapies, suggesting possibilities of potential interaction associated with vitamin B12 deficiencies.

### 3.4. Outcomes Reported for Monotherapies and Combination Therapies

The distributions of reported outcomes between monotherapies and combination therapies with metformin with vitamin B12 deficiencies are outlined in Table 4. Statistically significant differences were observed of increased hospitalization and life-threatening events were observed with metformin combination with pantoprazole compared to pantoprazole alone.

## 4. Discussion

### 4.1. Key Findings

This comprehensive pharmacovigilance analysis of the USFDA AERS database, encompassing over 29 million reports, revealed several critical findings regarding vitamin B12 deficiency associated with metformin and PPI use. First, both metformin and PPIs independently generated positive safety signals for vitamin B12 deficiency, confirming their individual risk profiles. Second, while combination therapies, particularly with pantoprazole, omeprazole, esomeprazole, and rabeprazole, demonstrated positive safety signals, the interaction analysis did not reach statistical significance. However, the elevated EBGM values observed with esomeprazole and rabeprazole combinations suggest potentially possible interaction effects warranting further investigation. Third, the clinical significance of these findings is underscored by the increased frequency of serious outcomes, including hospitalization and life-threatening events, particularly in patients receiving metformin–pantoprazole combination therapy. These findings are especially pertinent given the predominant elderly population in the reported cases; a demographic particularly vulnerable to vitamin B12 deficiency complications.

### 4.2. Comparison with Existing Literature

Our pharmacovigilance analysis has generated robust signals linking vitamin B12 deficiency with both metformin and PPI use independently, aligning with existing literature. A comprehensive primary care study demonstrated that metformin usage was the predominant factor associated with vitamin B12 deficiency, with higher daily doses significantly increasing the risk (OR: 2.79). Importantly, this risk is rarely isolated, patients with type 2 diabetes often have multiple comorbidities (e.g., renal impairment, cardiovascular disease) and polypharmacy exposure that may exacerbate nutrient malabsorption and confound the observed associations [22]. Such overlapping risk factors should be considered when interpreting our findings. Notably, patients receiving vitamin B12 supplementation showed substantially reduced odds of deficiency (OR: 0.37) [23]. While PPI use initially emerged as a significant risk factor in univariate analysis, its impact became statistically insignificant after multivariate adjustment, suggesting complex interactions between multiple risk factors.

Our study provides insights into the real-world implications of concurrent metformin and PPI use, suggesting potential interaction effects that warrant careful consideration. The predominance of elderly patients in our analysis is particularly concerning, given that this population already experiences vitamin B12 deficiency rates of 20–40% [24]. Elderly patients are additionally vulnerable due to frequent concomitant use of other agents, such as H2 blockers, antacids, and diuretics, that may independently impair absorption, alongside higher burden of chronic illness. These factors may amplify the deficiency risk observed with metformin and PPIs. The high prevalence of malabsorption in the elderly, affecting 40–70% of individuals [25], creates a perfect storm when combined with metformin and PPI therapy. This vulnerability is further complicated by the observation that while vitamin B12 deficiency typically develops after 5 years of metformin use, cases have been reported after just 3–4 months of therapy [26]. The clinical monitoring of vitamin B12 deficiency in elderly patients presents unique challenges. Common manifestations such as lethargy, weakness, dyspnea, and skin discoloration can be easily misattributed to normal aging processes. Therefore, we strongly advocate systematic laboratory monitoring of vitamin B12 levels, particularly in elderly patients receiving combination therapy [27]. This approach becomes crucial given the potential severity of outcomes observed in our analysis. While supplementation is a potential mitigation strategy, our focus should remain on risk identification and monitoring in patients with complex comorbidity and medication profiles. The presence of confounding factors, including nutritional status, alcohol intake, gastrointestinal disorders, and concurrent drug exposures, must be explored more explicitly in future research to clarify the independent and synergistic effects of metformin and PPIs. We have therefore streamlined supplementation-related discussions to emphasize its role only in high-risk groups, rather than detailing specific regimens.

Evidence supports proactive vitamin B12 supplementation in high-risk populations. A European Union Delphi consensus highlighted the cost-effectiveness of prophylactic supplementation compared to regular monitoring in elderly patients taking metformin or PPIs [28]. NHANES data revealed that while a daily vitamin B12 dose below 6 µg was sufficient for adult metformin users, elderly patients required significantly higher doses [29]. Research in older adults demonstrated that oral cyanocobalamin supplementation between 647 and 1032 μg achieved optimal reduction in methylmalonic acid concentrations, with required doses exceeding standard dietary recommendations by 200-fold [30]. High-dose oral supplementation (1–2 mg/day) has emerged as an effective alternative to parenteral administration in elderly individuals [24].

The development and validation of predictive tools for vitamin B12 deficiency risk remains an important area for future research. The metformin usage index, calculated as (daily dose × years of use)/1000, has shown promise as a predictor of cobalamin deficiency [31]. However, additional studies are needed to validate this and identify other predictive factors, particularly in the context of combination therapy with PPIs. Furthermore, research efforts should focus on determining optimal supplementation strategies, particularly in elderly populations receiving concurrent metformin and PPI therapy.

In terms of clinical practice recommendations, we advocate implementing mandatory baseline vitamin B12 screening before initiating combination therapy, followed by regular monitoring at 6-month intervals for the first two years and annually thereafter. This monitoring frequency should be increased in elderly patients or those with additional risk factors. Healthcare providers should consider prophylactic vitamin B12 supplementation (1–2 mg/day) in elderly patients receiving combination therapy, with dose adjustments based on regular monitoring results. The development of individualized monitoring plans based on patient-specific risk factors is crucial, as is the documentation and tracking of cumulative medication exposure using validated tools. At the healthcare system level, we recommend implementing automated alert systems in electronic health records to identify high-risk combinations and establish standardized protocols for vitamin B12 monitoring. Clear communication channels between primary care providers and specialists managing these patients should be established to ensure coordinated care. Additionally, future research should evaluate the cost-effectiveness of different monitoring approaches across various patient populations and healthcare settings. The development of evidence-based guidelines specific to combination therapy management, particularly in vulnerable populations, remains an important goal. These recommendations aim to optimize patient care while minimizing the risk of vitamin B12 deficiency-related complications in patients receiving concurrent metformin and PPI therapy.

### 4.3. Strengths and Limitations

Our study presents several notable strengths. First, this is one of the largest pharmacovigilance studies analyzing over 29 million reports to evaluate the combined risk of metformin and PPIs on vitamin B12 deficiency. The use of both frequentist and Bayesian approaches in signal detection enhances the robustness of our findings. The FAERS database’s extensive coverage and real-world setting provides insights into adverse events that might not be captured in controlled clinical trials. Additionally, our analysis of specific PPI–metformin combinations offers clinically relevant information for healthcare providers in selecting appropriate therapeutic combinations. The inclusion of clinical outcomes analysis, particularly focusing on serious adverse events such as hospitalizations and life-threatening events, provides valuable data for risk assessment in clinical practice.

Several limitations should be considered when interpreting our findings. The FAERS database is subject to inherent limitations of spontaneous reporting systems, including potential under-reporting, reporting bias, and the lack of a true denominator population. The absence of important clinical information such as medication doses, duration of therapy, and concurrent medications may affect the interpretation of causal relationships. Additionally, we could not account for over-the-counter PPI use or assess the impact of vitamin B12 supplementation on outcomes. The database also lacks information about potential confounding factors such as dietary habits, alcohol consumption, and other medical conditions that might affect vitamin B12 levels. Due to number constraints, we could not analyze the potential differences in the reporting rates between those with clinical impact of vitamin B12 deficiencies such as anemia and neuropathy. Furthermore, the predominance of reports from developed countries may limit the generalizability of our findings to other populations.

## 5. Conclusions

In conclusion, our comprehensive pharmacovigilance analysis demonstrates clear and consistent associations between vitamin B12 deficiency and the use of metformin and PPIs, both individually and in combination. The concurrent use of metformin and PPIs should be regarded as a red-flag scenario for vitamin B12 deficiency, particularly in elderly patients who are already at heightened risk. Specific PPI–metformin combinations revealed disproportionately elevated signals, underscoring the seriousness of this interaction. These findings provide compelling evidence for the mandatory implementation of proactive vitamin B12 monitoring protocols in patients prescribed both agents, with a lower threshold for intervention in older adults and those with polypharmacy or chronic comorbidities. Prophylactic vitamin B12 supplementation should be strongly considered in high-risk populations as part of routine care. While prospective studies are warranted to refine optimal monitoring and supplementation strategies, our results highlight the immediate need for heightened clinical vigilance to prevent vitamin B12 deficiency-related complications in patients receiving concurrent metformin–PPI therapy.

## Figures and Tables

**Table 1:** Demographic characteristics of patients included in the reports.

Characteristics.		Metformin Monotherapy (*n* = 274)	Metformin Combination Therapies with PPIs (*n* = 88)				
			Pantoprazole (*n* = 30)	Esomeprazole (*n* = 18)	Lansoprazole (*n* = 3)	Omeprazole (*n* = 33)	Rabeprazole (*n* = 4)
Age groups [*n* (%)]	<18 years	2 (0.7)	Nil	Nil	Nil	Nil	Nil
	≥18 to <40 years	13 (4.7)		4 (22.2)			
	≥40 to <65 years	56 (20.4)	2 (6.7)	3 (16.7)		8 (24.2)	
	≥65 years	154 (56.2)	22 (73.3)	10 (55.6)	2 (66.7)	20 (60.6)	4 (100)
	Not specified	49 (17.9)	6 (20)	1 (5.6)	1 (33.3)	5 (15.2)	Nil
Quantitative age (years)	Mean (SD)	66.1 (15.8)	70.6 (9.3)	59.8 (16.1)	77 (9.9)	68.1 (14.4)	82 (8)
	Median (range)	70 (15–90)	73 (40–82)	66 (34–86)	77 (70–84)	70 (43–81)	86 (70–86)
Gender [*n* (%)]	Male	122 (44.5)	13(43.3)	9 (50)	1 (33.3)	13 (39.4)	3 (75)
	Female	121 (44.2)	14 (46.7)	9 (50)	2 (66.7)	18 (54.5)	Nil
	Unknown	29 (11.3)	3 (10)	Nil	Nil	2 (6.1)	1 (25)
Reporting year [*n* (%)]	2004–2008	29 (10.6)	Nil		1 (33.3)	2 (6.1)	1 (25)
	2009–2012	19 (6.9)	5 (16.7)	3 (16.7)	Nil	5 (15.2)	Nil
	2013–2016	33 (12)	6 (20)	5 (83.3)		2 (6.1)	
	2017–2020	136 (49.6)	9 (30)	Nil		17 (51.5)	3 (75)
	2021–2024 (June)	57 (20.8)	10 (33.3)	10 (55.6)	2 (66.7)	7 (21.2)	Nil
Reporting top countries	USA	53 (19.3)	6 (20)	5 (27.8)	1 (33.3)	8 (24.2)	
	Other countries and not reported	221 (80.7)	24 (80)	13 (72.2)	2 (66.7)	25 (75.8)	4 (100)

USA: The United States of America.

**Table 2:** Signal detection measures for the risk of vitamin B12 deficiency.

Drugs	PRR	Lower 95% CI of PRR	Upper 95% CI of PRR	RRR	χ^2^	Number of Reports	IC025	EBGM05
Monotherapy								
Metformin	24.5	21.6	27.7	21.9	5474.5	274	3.9	19.3
Pantoprazole	14.6	11.3	18.8	14.3	740.8	61	3	11.1
Omeprazole	10.1	7.5	13.5	9.9	351.4	45	2.5	7.4
Esomeprazole	7.4	5.7	9.6	7.2	295	56	2.2	5.5
Rabeprazole	9.1	3.8	21.9	9.1	28.4	5	1.3	3.8
Lansoprazole	3.9	2.4	6.3	3.9	33.6	17	1.2	2.4
Dexlansoprazole	6.1	2.8	13.7	6.1	20.9	6	1.2	2.7
Combination therapies with metformin								
Pantoprazole	11.1	7.8	16	11	263.8	30	2.4	7.7
Omeprazole	10	7.1	14.1	9.9	255.1	33	2.3	7
Esomeprazole	15.3	9.6	24.4	15.2	224.8	18	2.5	9.5
Rabeprazole	12.3	4.6	32.7	12.3	30.9	4	1.4	4.6
Lansoprazole	2.5	0.8	7.8	2.5	1.4	3	0.4	0.8

RRR: Relative reporting ratio; PRR: Proportional reporting ratio; χ^2^: Chi-square test statistics; IC: Information component; and EBGM: Empirical Bayes geometric mean. Metformin showed the strongest and most consistent association with vitamin B12 deficiency among all monotherapies. Among PPIs, pantoprazole and omeprazole demonstrated notable signals, with pantoprazole having the highest strength. Combination therapies generally produced higher signal strengths compared to PPI monotherapy, particularly with esomeprazole and rabeprazole, suggesting a potential additive or synergistic risk. The metformin–pantoprazole combination was especially important, as it not only showed stronger reporting signals but was also associated with worse clinical outcomes such as hospitalization and life-threatening events. In contrast, lansoprazole combinations demonstrated weaker or inconsistent signals, indicating possible variation in risk across different PPIs.

**Table 3:** Interaction signal scores for the risk of vitamin B12 deficiency with metformin, PPIs and the combination therapies.

Drugs	EBGM	EB05	EB95	INTSS
Monotherapy				
Metformin	21.9	19.7	24.3	NA
Pantoprazole	14.3	11.5	17.7	
Omeprazole	9.9	7.7	12.7	
Esomeprazole	7.2	5.8	9	
Rabeprazole	9.1	4.3	19	
Lansoprazole	3.9	2.6	5.8	
Dexlansoprazole	6.1	3.1	12	
Combination therapies with metformin				
Pantoprazole	11	8.1	14.9	0.3
Omeprazole	9.9	7.4	13.2	0.3
Esomeprazole	15.2	10.3	22.4	0.4
Rabeprazole	12.3	5.4	28	0.2
Lansoprazole	2.5	1	6.5	0.2

EBGM: Empirical Bayes geometric mean; EB05: Lower limit of 90% CI of EBGM; EB95: Upper limit of 90% CI of EBGM; INTSS: Interaction signal score; and NA: Not applicable. Metformin showed the strongest signal for vitamin B12 deficiency among all drugs analyzed. Pantoprazole and omeprazole demonstrated moderate signal strengths, while esomeprazole and rabeprazole also showed positive associations though with wider variability. When combined with metformin, esomeprazole and rabeprazole produced higher reporting signals than their monotherapies, suggesting a possible additive effect, although the interaction scores (INTSS) did not reach statistical significance. The metformin–pantoprazole and metformin–omeprazole combinations also showed increased reporting of B12 deficiency but again without strong interaction evidence. Lansoprazole combinations consistently yielded weaker signals, indicating potential differences in risk across PPIs. Overall, these findings suggest that while both metformin and PPIs independently increase the likelihood of vitamin B12 deficiency, certain combinations, particularly with esomeprazole and rabeprazole, may warrant closer clinical attention despite the absence of statistically significant interaction signals.

**Table 4:** Reported outcomes between the interventions.

Drugs	Hospitalization	Life-Threatening Events	Disability	χ^2^; df; *p*-Value
Metformin	145	15	15	NA
Pantoprazole	13	4	6	7.2; 2; 0.03 *
Metformin/Pantoprazole	19	5	0	
Omeprazole	12	1	2	5.2; 2; 0.07
Metformin/Omeprazole	24	0	0	
Esomeprazole	14	0	2	Not estimable
Metformin/Esomeprazole	15	0	0	
Rabeprazole	1	1	2	3.9; 2; 0.1
Metformin/Rabeprazole	3	0	0	
Lansoprazole	3	0	5	Not estimable
Metformin/Lansoprazole	1	0	1	
Dexlansoprazole	0	0	2	NA

χ^2^: Chi-square test statistics; df: degree of freedom; *—Statistically significant; and NA: Not applicable.

## Data Availability

The datasets generated during and/or analyzed during the current study are publicly available from the USFDA AERS database.

## Supplementary Materials

The following supporting information can be downloaded at: https://www.mdpi.com/article/10.3390/diseases13100334/s1, Table S1: Search strategy used in this study.
