# Supplementary material for: Vitamin B12 Deficiency Associated with Metformin and Proton Pump Inhibitors and Their Combinations: Results from a Disproportionality and Interaction Analysis
Source: Diseases. 2025 Oct 10;13(10):334. doi: 10.3390/diseases13100334 (PMC12562576; doi:10.3390/diseases13100334)
Supplement: Supplementary file 1 [file diseases-13-00334-s001.zip › diseases-3837504-supplementary.pdf]

Electronic Supplementary Table S1. Search strategy used in this study.

Drug/Drug combinations	Search terms
Metformin	**Drug:** Metformin AND NOT Pantoprazole AND NOT Omeprazole AND NOT Esomeprazole AND NOT Lansoprazole AND NOT Dexlansoprazole AND NOT Rabeprazole.<br><br>**Role:** Primary suspect<br><br>**Adverse event preferred Term:** Vitamin B12 deficiency OR Vitamin B12 decreased OR Anaemia vitamin B12 deficiency OR Neuropathy vitamin B12 deficiency.
Pantoprazole	**Drug:** Pantoprazole AND NOT Metformin<br><br>**Role:** Primary suspect<br><br>**Adverse event preferred Term:** Vitamin B12 deficiency OR Vitamin B12 decreased OR Anaemia vitamin B12 deficiency OR Neuropathy vitamin B12 deficiency.
Omeprazole	**Drug:** Omeprazole AND NOT Metformin<br><br>**Role:** Primary suspect<br><br>**Adverse event preferred Term:** Vitamin B12 deficiency OR Vitamin B12 decreased OR Anaemia vitamin B12 deficiency OR Neuropathy vitamin B12 deficiency.
Esomeprazole	**Drug:** Esomeprazole AND NOT Metformin<br><br>**Role:** Primary suspect<br><br>**Adverse event preferred Term:** Vitamin B12 deficiency OR Vitamin B12 decreased OR Anaemia vitamin B12 deficiency OR Neuropathy vitamin B12 deficiency.
Lansoprazole	**Drug:** Lansoprazole AND NOT Metformin<br><br>**Role:** Primary suspect<br><br>**Adverse event preferred Term:** Vitamin B12 deficiency OR Vitamin B12 decreased OR Anaemia vitamin B12 deficiency OR Neuropathy vitamin B12 deficiency.
Dexlansoprazole	**Drug:** Dexlansoprazole AND NOT Metformin<br><br>**Role:** Primary suspect

	**Adverse event preferred Term:** Vitamin B12 deficiency OR Vitamin B12 decreased OR Anaemia vitamin B12 deficiency OR Neuropathy vitamin B12 deficiency.
Rabeprazole	**Drug:** Rabeprazole AND NOT Metformin<br><br>**Role:** Primary suspect<br><br>**Adverse event preferred Term:** Vitamin B12 deficiency OR Vitamin B12 decreased OR Anaemia vitamin B12 deficiency OR Neuropathy vitamin B12 deficiency.
Metformin and Pantoprazole	**Drug:** Metformin AND Pantoprazole<br><br>**Role:** Any role.<br><br>**Adverse event preferred Term:** Vitamin B12 deficiency OR Vitamin B12 decreased OR Anaemia vitamin B12 deficiency OR Neuropathy vitamin B12 deficiency.
Metformin and Omeprazole	**Drug:** Metformin AND Omeprazole<br><br>**Role:** Any role.<br><br>**Adverse event preferred Term:** Vitamin B12 deficiency OR Vitamin B12 decreased OR Anaemia vitamin B12 deficiency OR Neuropathy vitamin B12 deficiency.
Metformin and Esomeprazole	**Drug:** Metformin AND Esomeprazole<br><br>**Role:** Any role.<br><br>**Adverse event preferred Term:** Vitamin B12 deficiency OR Vitamin B12 decreased OR Anaemia vitamin B12 deficiency OR Neuropathy vitamin B12 deficiency.
Metformin and Rabeprazole	**Drug:** Metformin AND Rabeprazole<br><br>**Role:** Any role.<br><br>**Adverse event preferred Term:** Vitamin B12 deficiency OR Vitamin B12 decreased OR Anaemia vitamin B12 deficiency OR Neuropathy vitamin B12 deficiency.
Metformin and Lansoprazole	**Drug:** Metformin AND Lansoprazole<br><br>**Role:** Any role.<br><br>**Adverse event preferred Term:** Vitamin B12 deficiency OR Vitamin B12 decreased OR Anaemia vitamin B12 deficiency OR Neuropathy vitamin B12 deficiency.

Metformin and Dexlansoprazole	**Drug:** Metformin AND Dexlansoprazole<br><br>**Role:** Any role.<br><br>**Adverse event preferred Term:** Vitamin B12 deficiency OR Vitamin B12 decreased OR Anaemia vitamin B12 deficiency OR Neuropathy vitamin B12 deficiency.
